# Supplementary material for: RTCB Complex Regulates Stress-Induced tRNA Cleavage
Source: Int J Mol Sci. 2022 Oct 28;23(21):13100. doi: 10.3390/ijms232113100 (PMC9655011; doi:10.3390/ijms232113100)
Supplement: Supplementary file 1 [file ijms-23-13100-s001.zip › ijms-1965014-supplementary.pdf]

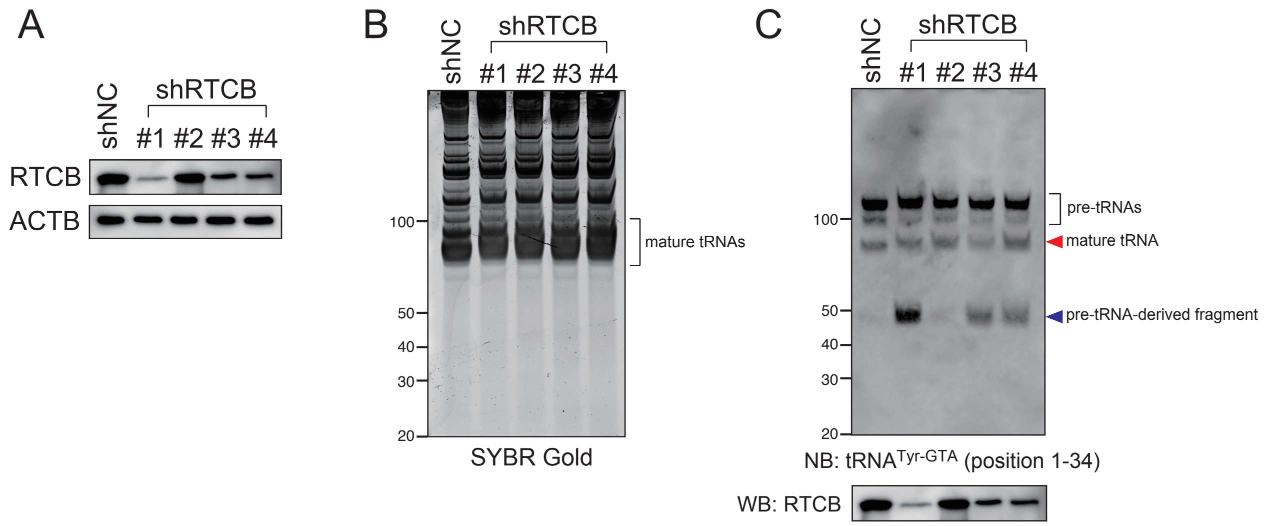

Figure S1. The effect of RTCB knockdown at day 7 on the amount of pre-tRNA^{Tyr}-derived fragment. (A) Knockdown efficiency of four constructs at day 7. (B) SYBR Gold staining of total RNAs. (C) Norther blotting for tRNA$^{Tyr\text{-}GTA}$. Note that the amount of pre-tRNA^{Tyr}-derived fragment is in proportion to knockdown efficiency of RTCB.

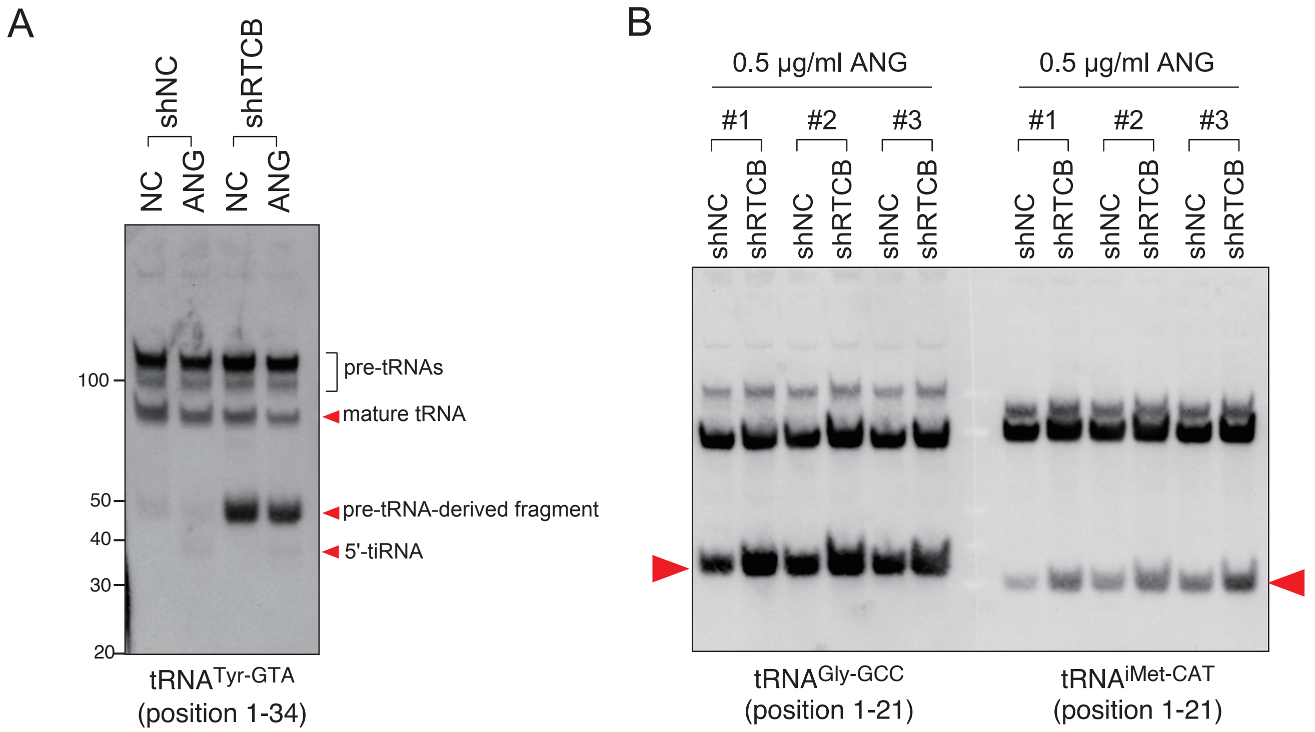

Figure S2. Additional data related to Figure 1A-C. (A) Northern blotting for tRNA$^{Tyr\text{-}GTA}$. (B) Raw image of Northern blotting for quantification of ANG-induced 5'-tiRNAs. 5'-tiRNAs are indicated by red arrowheads.

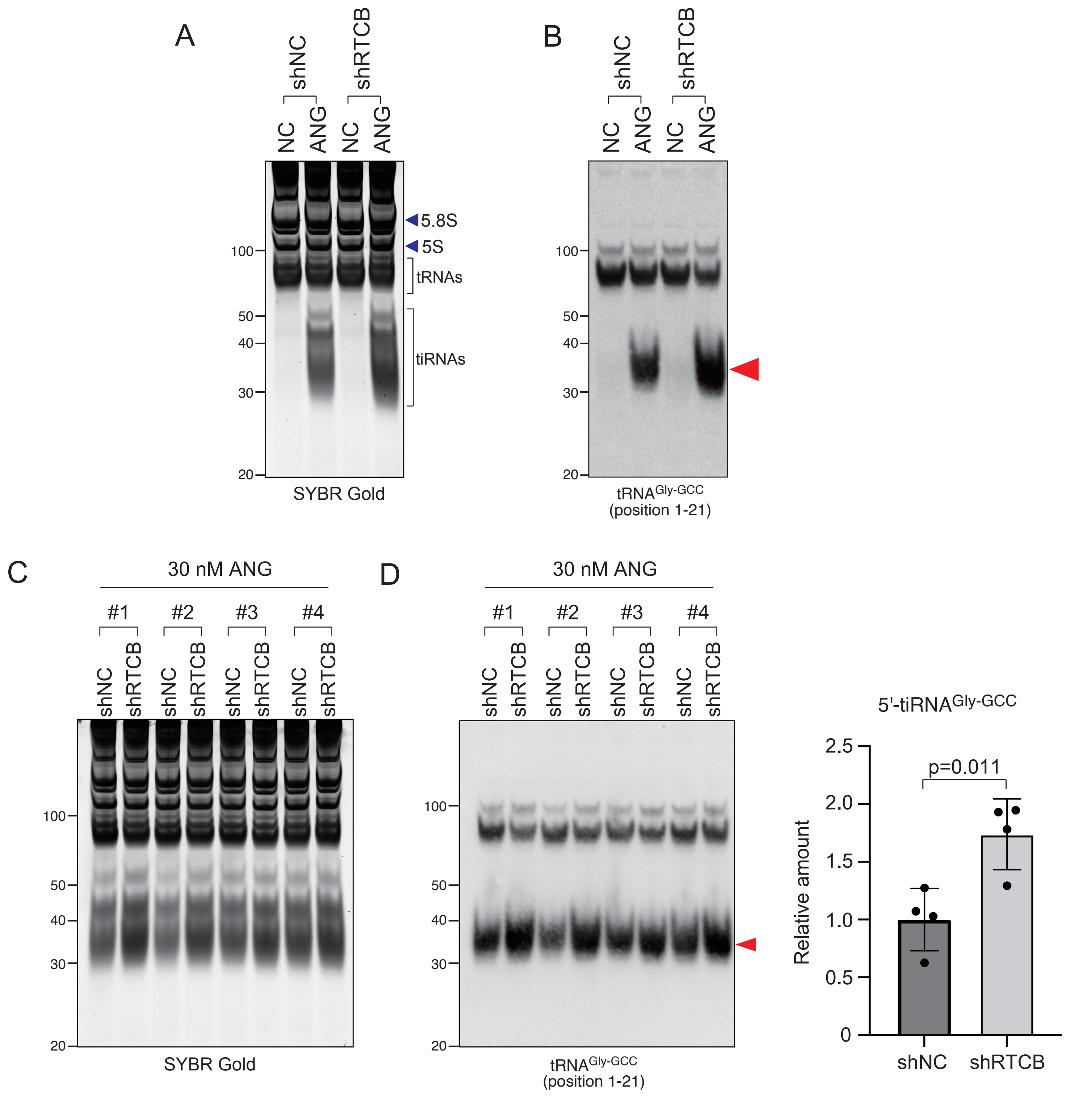

Figure S3. RTCB knockdown enhances tiRNA production induced by *in lysate* ANG digestion. (A) SYBR Gold staining and (B) Northern blotting for $tRNA^{Gly\text{-}GCC}$. $5'\text{-}tiRNA^{Gly\text{-}GCC}$ is indicated by a red arrowhead. (C-D) Raw images used for quantification of $5'\text{-}tiRNA^{Gly\text{-}GCC}$. (C) SYBR Gold staining and (D) Northern blotting for $tRNA^{Gly\text{-}GCC}$. $5'\text{-}tiRNA^{Gly\text{-}GCC}$ is indicated by a red arrowhead. Relative amount of $5'\text{-}tiRNA^{Gly\text{-}GCC}$ calculated by densitometry is also shown (n=4 each).

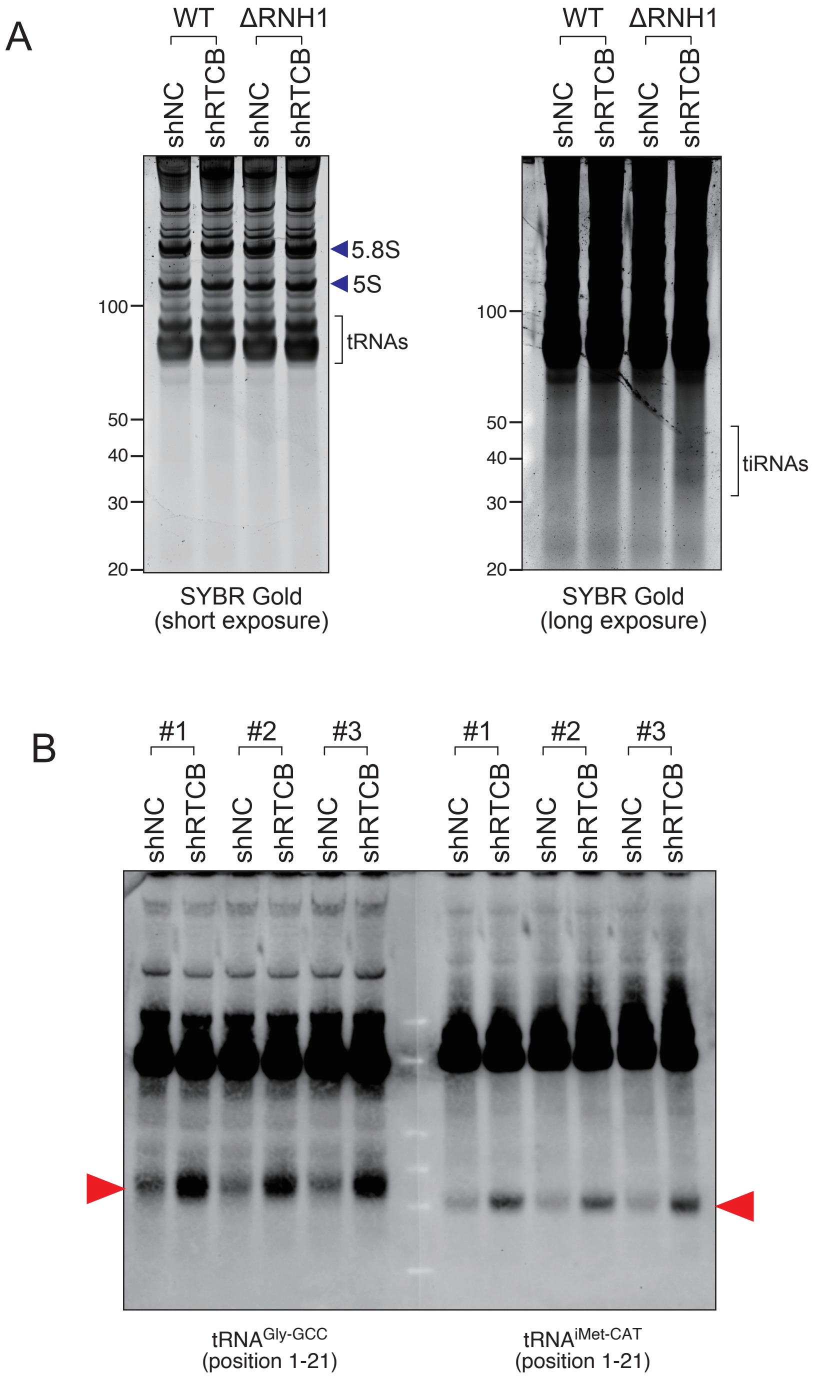

Figure S4. Additional data for Figure 1D. RTCB knockdown increases constitutively produced tRNAs in RNH1 knockout cells. (A) SYBR Gold staining. (B) Raw images of Northern blotting for quanitification of 5'-tiRNAs. 5'-tiRNAs are indicated by red arrowheads.

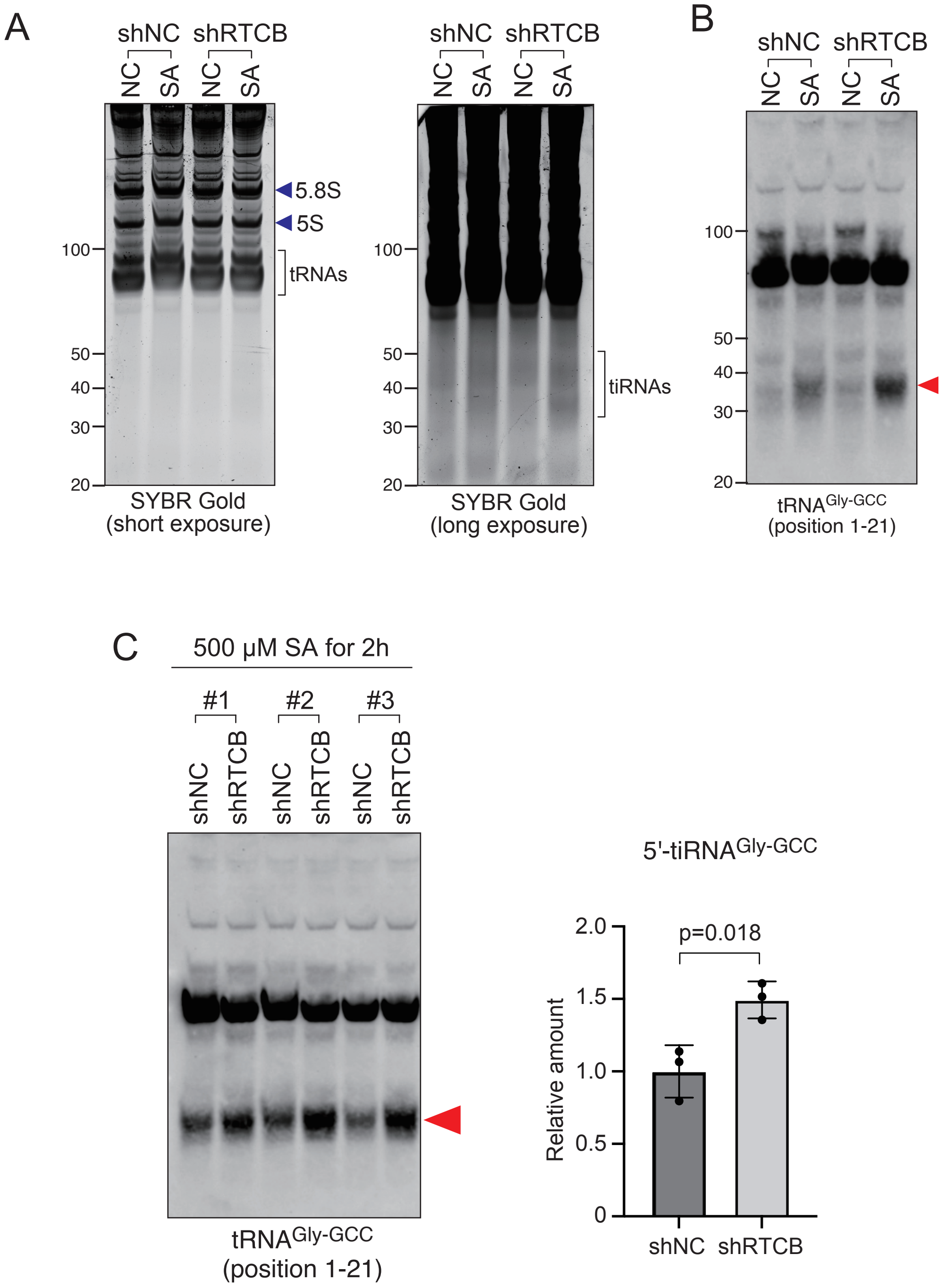

Figure S5. RTCB knockdown enhances sodium arsenite-induced tiRNA production. (A) SYBR Gold staining and (B) Northern blotting for tRNA$^{Gly\text{-}GCC}$. 5'-tiRNA$^{Gly\text{-}GCC}$ is indicated by a red arrowhead.

(C) Raw images for quantification of 5'-tiRNA$^{Gly\text{-}GCC}$ (indicated by a red arrowhead). Relative amount of 5'-tiRNA$^{Gly\text{-}GCC}$ calculated by densitometry is also shown (n=3 each). SA, sodium arsenite.

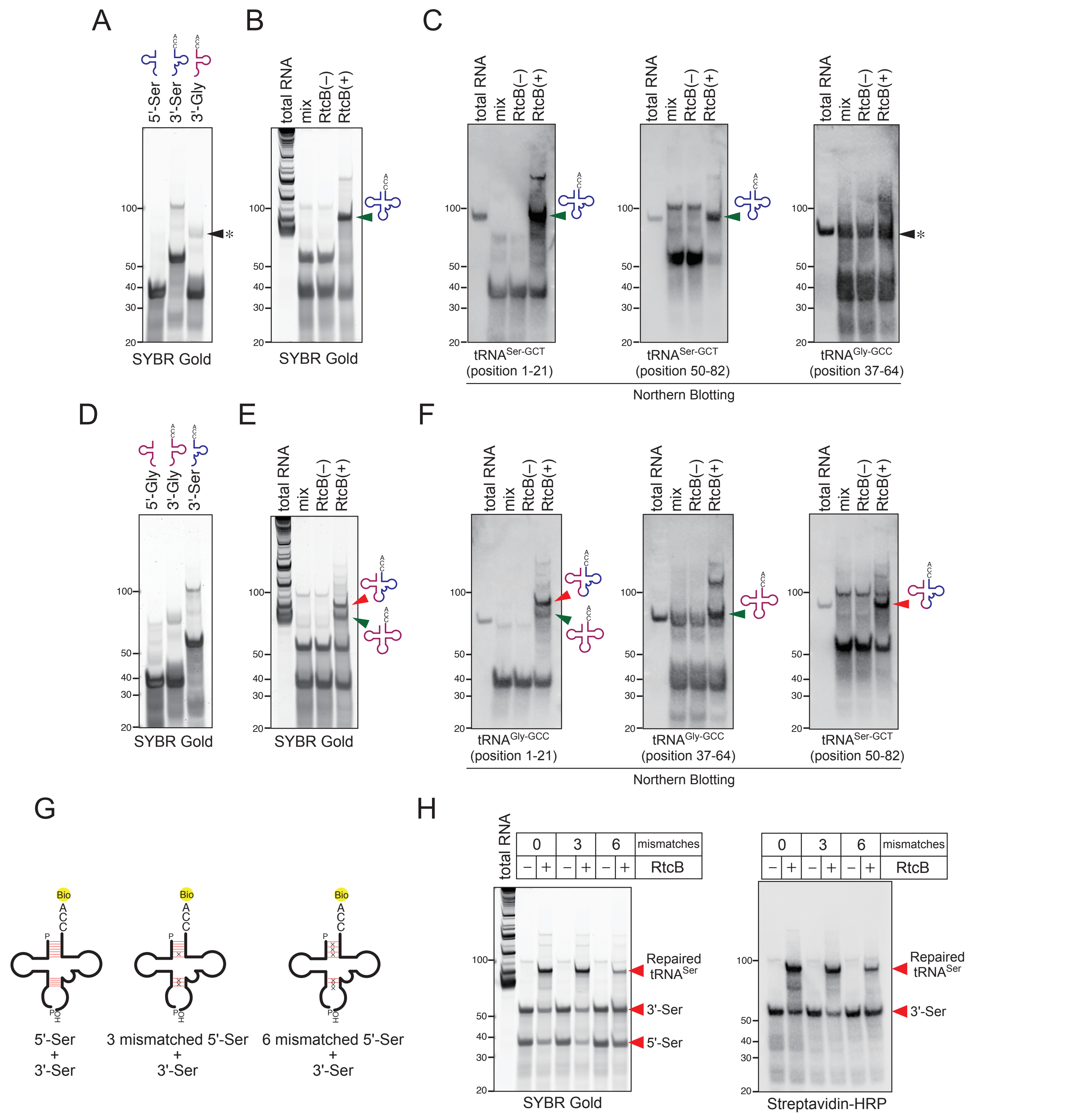

Figure S6. RtcB can generate chimeric tRNAs *in vitro*. (A-C) 5'-tiRNA^{Ser} is preferentially ligated to 3'-tiRNA^{Ser} compared to 3'-tiRNA^{Gly}. (A) SYBR Gold staining of synthetic oligos. (B) SYBR Gold staining and (C) Northern blotting of ligation products. Asterisk suggests a dimer of 3'-tiRNA^{Gly}. (D-F) 5'-tiRNA^{Gly} is nonspecifically ligated to both 3'-tiRNA^{Gly} and 3'-tiRNA^{Ser}. (D) SYBR Gold staining of synthetic oligos. (E) SYBR Gold staining and (F) Northern blotting of ligation products. (G-H) Mismatches within the stem structure decrease ligation efficiency. (G) Schema of combination of oligos. Positions of mutations in 5'-tiRNAs^{Ser} are indicated as “x” . (H) Ligation products are visualized by SYBR Gold staining and Streptavidin-HRP system.

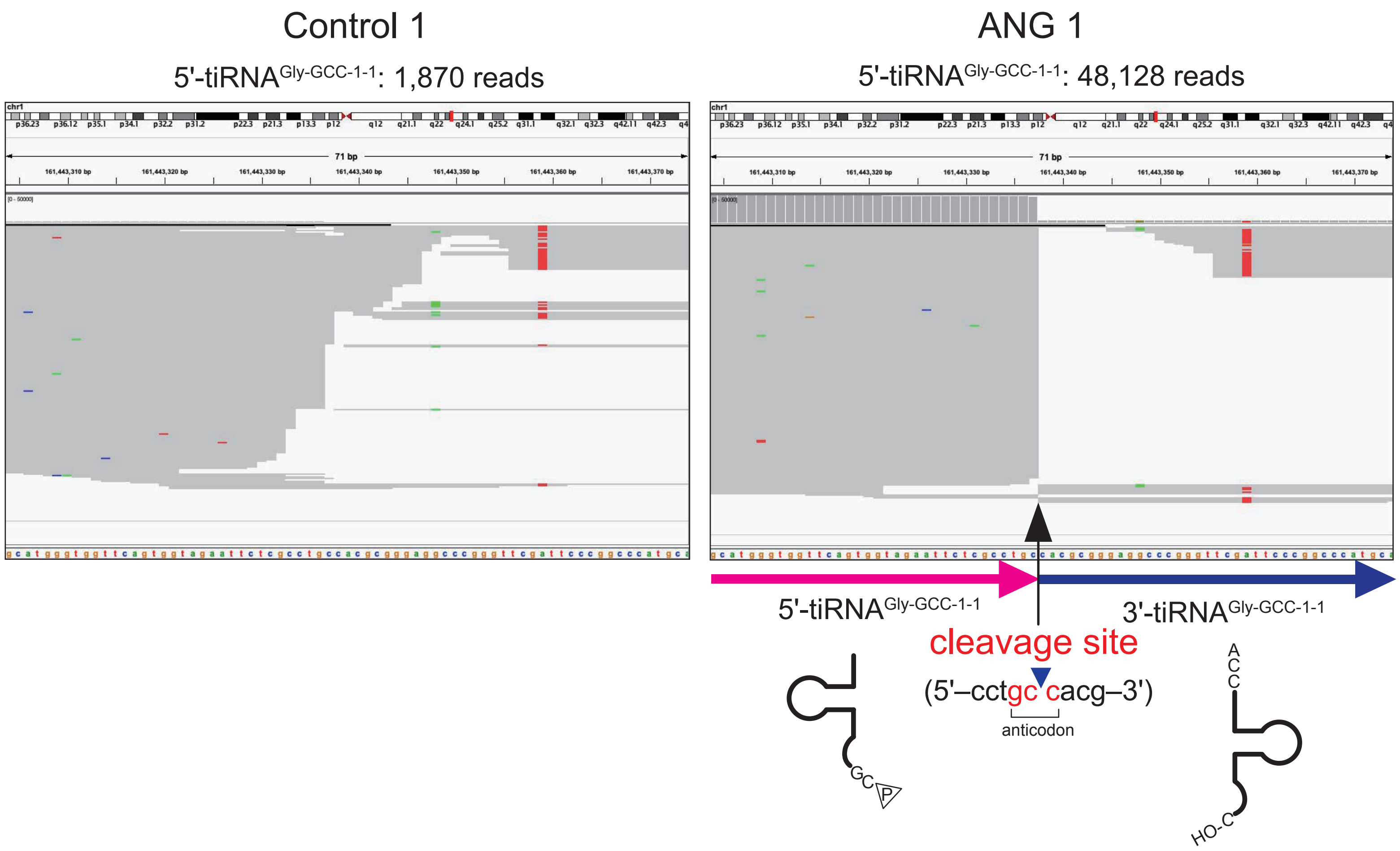

Figure S7. Identification of the cleavage site by ANG in tRNA$^{Gly\text{-}GCC\text{-}1}$. The reads mapped to tRNA$^{Gly\text{-}GCC\text{-}1\text{-}1}$ gene were visualized by Intergated Genome Viewer (IGV). ANG treatment significantly increased 34-nt 5'-tiRNA$^{Gly\text{-}GCC\text{-}1\text{-}1}$, suggesting that ANG cleaves between c and c in the anticodon loop of tRNA$^{Gly\text{-}GCC\text{-}1}$.

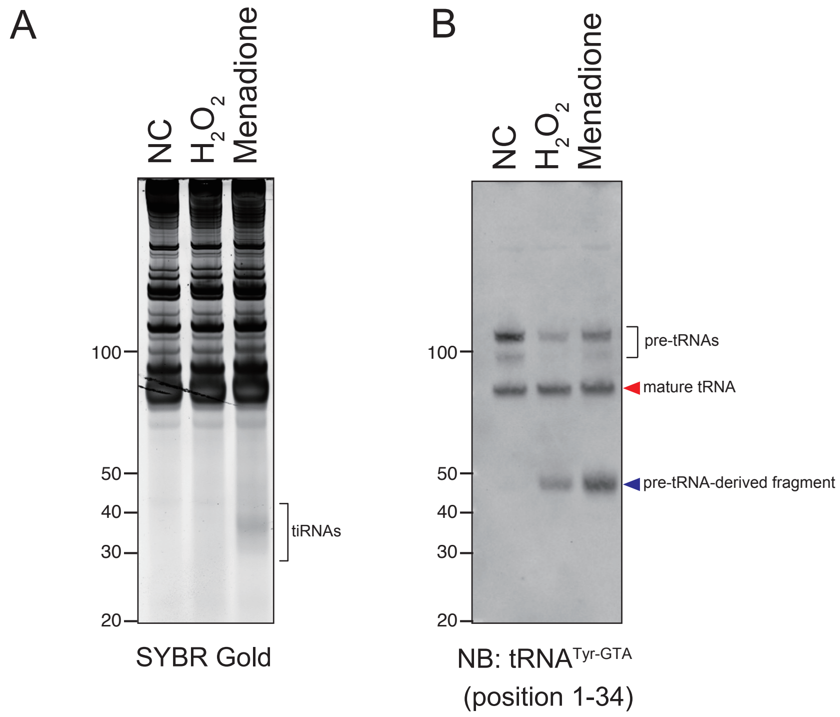

Figure S8. Both H_2O_2 and Menadione induce pre-tRNA^{Tyr}-derived fragment through inhibition of RTCB. treatment enhances tiRNA production in RNH1 knockout cells. (A) SYBR Gold staining and (B) Northern blotting for tRNA$^{Tyr-GTA}$.

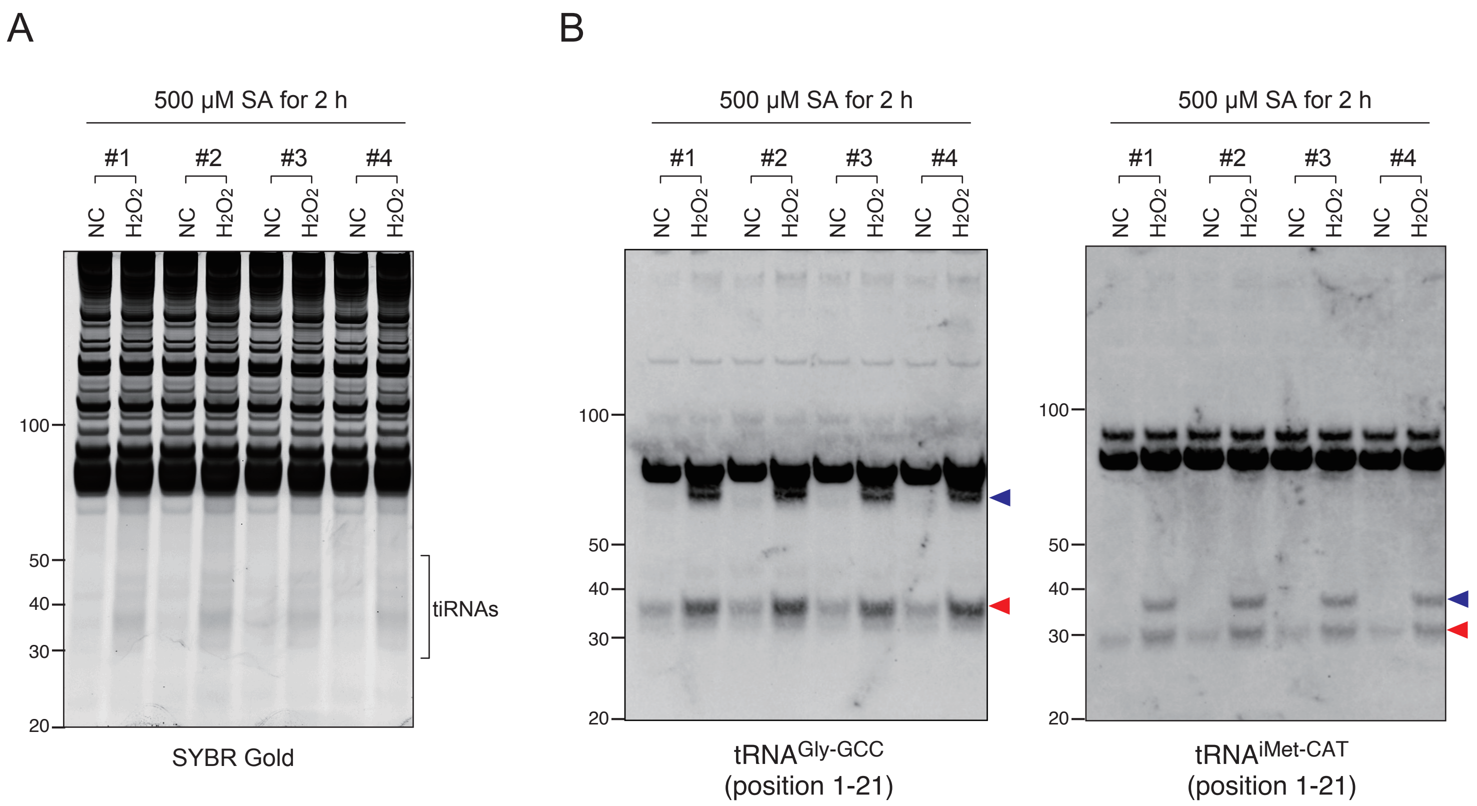

Figure S9. Raw images for quantification of 5'-tiRNAs related to Figure 4A-C. (A) SYBR Gold staining and (B) Northern blotting for $tRNA^{Gly\text{-}GCC}$ and $tRNA^{iMet\text{-}CAT}$. Canonical 5'-tiRNAs are indicated by red arrowheads, while non-canonical bands detected only by combination of sodium arsenite and H_2O_2 are indicated by blue arrowheads. SA, sodium arsenite.

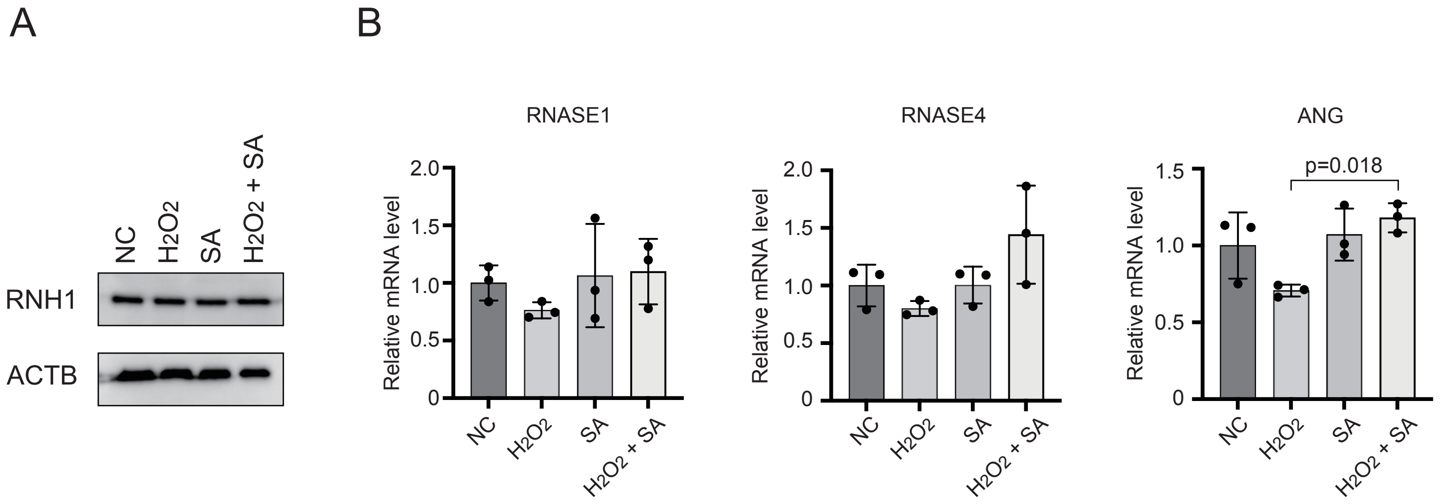

Figure S10. The effect of stress stimuli on the expression of RNH1 and RNases (related to Figure 4A-B). (A) Western blotting for RNH1. Two hour treatment with H_2O_2 and/or sodium arsenite (SA) does not change the expression levels of RNH1 proteins. (B) mRNA expression levels of RNASE1, RNASE4 and angiogenin (ANG). Relative expression levels to GAPDH mRNA are shown. SA, sodium arsenite.

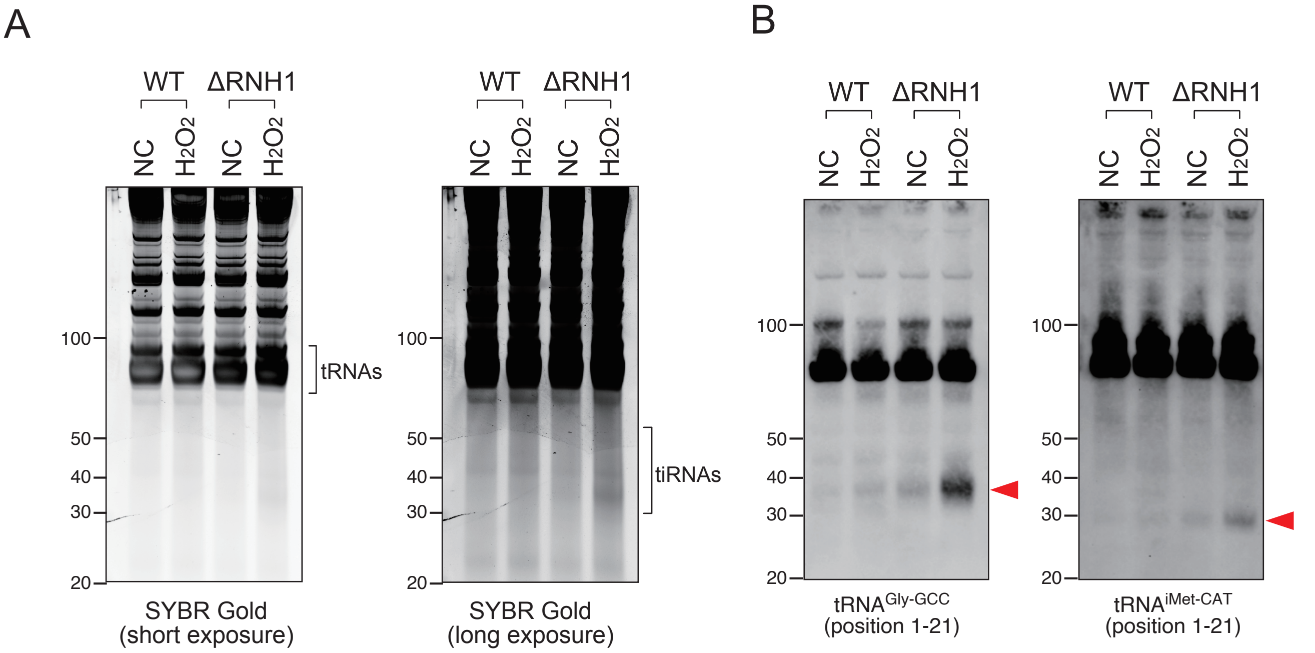

Figure S11. H_2O_2 treatment enhances tiRNA production in RNH1 knockout cells. (A) SYBR Gold staining and (B) Northern blotting for $tRNA^{Gly\text{-}GCC}$ and $tRNA^{iMet\text{-}CAT}$. 5'-tiRNAs are indicated by red arrowheads.

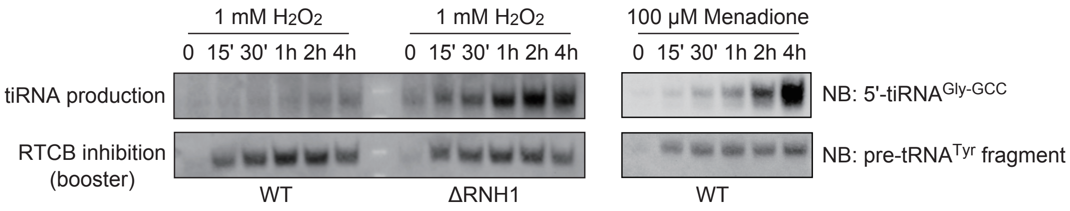

Figure S12. Time courses of tiRNA production and RTCB inhibition under oxidative stress. tiRNA production and RTCB inhibition were assessed by the amounts of 5'-tiRNA$^{Gly\text{-}GCC}$ and pre-tRNA^{Tyr}-derived fragment, respectively.

		Sequences
shNC(negative control)	top strand	ccggGCATTCACTTGGATAGTAActcgagTTACTATCCAAGTGAATGCttttt
	bottom strand	aattaaaaaGCATTCACTTGGATAGTAActcgagTTACTATCCAAGTGAATGC
shRTCB #1	top strand	ccggGGAATTGTTCATCGATCTActcgagTAGATCGATGAACAATTCCttttt
	bottom strand	aattaaaaaGGAATTGTTCATCGATCTActcgagTAGATCGATGAACAATTCC
shRTCB #2	top strand	ccggGATATGGGTTTGCTATTGGctcgagCCAATAGCAAACCCATATCttttt
	bottom strand	aattaaaaaGATATGGGTTTGCTATTGGctcgagCCAATAGCAAACCCATATC
shRTCB #3	top strand	ccggGGTGTCCGCTTGCTAAGAActcgagTTCTTAGCAAGCGGACACCttttt
	bottom strand	aattaaaaaGGTGTCCGCTTGCTAAGAActcgagTTCTTAGCAAGCGGACACC
shRTCB #4	top strand	ccggGAACGGACACTGTTAGTACctcgagGTACTAACAGTGTCCGTTCttttt
	bottom strand	aattaaaaaGAACGGACACTGTTAGTACctcgagGTACTAACAGTGTCCGTTC

Table S1. The sequences of DNA oligos inserted to pLKO1 vector for shRNA- mediated knockdown.

Probes	Sequences
$tRNA^{Tyr-GTA}$ (position 1-34)	5'-CAGTCCTCCGCTCTACCAACTGAGCTATCGAAGG-3'
$tRNA^{Gly-GCC}$ (position 1-21)	5'-CTACCACTGAACCACCCATGC-3'
$tRNA^{Gly-GCC}$ (position 37-64)	5'-GCCGGGAATCGAACCCGGGCCTCCCGCG-3'
$tRNA^{iMet-CAT}$ (position 1-21)	5'-CTTCCGCTGCGCCACTCTGCT-3'
$tRNA^{Ser-GCT}$ (position 1-21)	5'-TAACCACTCGGCCACCTCGTC-3'
$tRNA^{Ser-GCT}$ (position 50-82)	5'-CGACGAGGGTGGGATTCGAACCCACGCGTGCAG-3'

Table S2. The sequences of DNA oligo probes for Northern blotting used in this study.

RNA oligos	Sequences
5'-tRNA^{Ser}	/5Phos/GACGAGGUGGCCGAGUGGUUAAGGCGAUGGACUGC/3Phos/
3'-tiRNA^{Ser}	UAAUCCAUUGUGCUCUGCACGCGUGGGUUCGAAUCCCACCCUCGUCGCCA/3Bio/
5'-tRNA^{Gly}	/5Phos/GCAUGGGUGGUUCAGUGGUAGAAUUCUCGCCUGC/3Phos/
3'-tRNA^{Gly}	CACGCGGGAGGCCCGGGUUCGAUUCCCGGCCCAUGCACCA/3Bio/
3-mismatched 5'-tiRNA^{Ser}	/5Phos/GACGACGUGGCCGAGUGGUUAAGGCGAAGCACUGC/3Phos/
6-mismatched 5'-tiRNA^{Ser}	/5Phos/GUCCACGUGGCCGAGUGGUUAAGGCGAAGCUCUGC/3Phos/

Table S3. The sequences of synthetic tiRNAs used in this study. The positions of mutations in mismatch-containing 5'-tiRNAs^{Ser} are indicated in red. Note that 5'-tiRNAs and 3'-tiRNAs possess 3'-phosphate and 5'-OH, respectively, so that RtcB can ligate them. 5Phos, 5'-phosphorylated; 3Phos, 3'-phosphorylated; 3Bio, 3'-biotinylated.
